# A Statistical Thermodynamic Model for Ligands Interacting With Ion Channels: Theoretical Model and Experimental Validation of the KCNQ2 Channel

**DOI:** 10.3389/fphar.2018.00150

**Published:** 2018-03-09

**Authors:** Fang Bai, Xiaoping Pi, Ping Li, Pingzheng Zhou, Huaiyu Yang, Xicheng Wang, Min Li, Zhaobing Gao, Hualiang Jiang

**Affiliations:** ^1^Department of Engineering Mechanics, State Key Laboratory of Structural Analysis for Industrial Equipment, and Faculty of Chemical, Environmental, and Biological Science and Technology, Dalian University of Technology, Dalian, China; ^2^Drug Discovery and Design Center, State Key Laboratory of Drug Research, CAS Key Laboratory of Receptor Research, Shanghai Institute of Materia Medica, Chinese Academy of Sciences, Shanghai, China; ^3^University of Chinese Academy of Sciences, Beijing, China; ^4^Department of Neuroscience, Johns Hopkins University, Baltimore, MD, United States

**Keywords:** KCNQ2 potassium channel, hill equation, thermodynamic model, patch clamp electrophysiology, activators

## Abstract

Ion channels are important therapeutic targets, and their pharmacology is becoming increasingly important. However, knowledge of the mechanism of interaction of the activators and ion channels is still limited due to the complexity of the mechanisms. A statistical thermodynamic model has been developed in this study to characterize the cooperative binding of activators to ion channels. By fitting experimental concentration-response data, the model gives eight parameters for revealing the mechanism of an activator potentiating an ion channel, i.e., the binding affinity (*K*_*A*_), the binding cooperative coefficients for two to four activator molecules interacting with one channel (γ, μ, and ν), and the channel conductance coefficients for four activator binding configurations of the channel (*a, b, c*, and *d*). Values for the model parameters and the mechanism underlying the interaction of ztz240, a proven KCNQ2 activator, with the wild-type channel have been obtained and revealed by fitting the concentration-response data of this activator potentiating the outward current amplitudes of KCNQ2. With these parameters, our model predicted an unexpected bi-sigmoid concentration-response curve of ztz240 activation of the WT-F137A mutant heteromeric channel that was in good agreement with the experimental data determined in parallel in this study, lending credence to the assumptions on which the model is based and to the model itself. Our model can provide a better fit to the measured data than the Hill equation and estimates the binding affinity, as well as the cooperative coefficients for the binding of activators and conductance coefficients for binding states, which validates its use in studying ligand-channel interaction mechanisms.

## Introduction

The KCNQ family of potassium channels, a type of ion channel, is widely expressed in different tissues (Brown, 2008). There are five isoforms with more than 40% amino acid identity within the six-transmembrane segment core regions. These channels are activated at sub-threshold membrane potentials. Because activation of these channels could dampen membrane excitability, potentiation of these channels by synthetic chemicals is believed to be beneficial in treating diseases with hyperexcitability, such as epilepsy and neuropathic pain (Surti et al., 2005; Lawson and Mckay, 2006). A number of compounds have been reported to display potentiation activities on KCNQ channels (Xiong et al., 2008). For example, potent anti-epileptic activities were reported for *N*-(6-chloro-pyridin-3-yl)-3,4-difluoro-benzamide (ICA-27243) in various rodent convulsant models, and a KCNQ2 activator, Retigabine, was approved for treatment of human epilepsy by the FDA in 2011 (Padilla et al., 2009; Stafstrom et al., 2011). In an effort to examine the chemical repertoire of KCNQ activators, Li's lab screened a collection of 20,000 compounds against KCNQ2 homomeric channels and identified multiple series of structures with distinct chemotypes. *N*-(6-chloro-pyridin-3-yl)-4-fluorobenzamide (ztz240) is one of the potent activators of KCNQ2 produced from this screening enterprise (Gao et al., 2010). Through mutagenesis and electrophysiological determination in conjunction with molecular modeling and simulation, we have identified the binding sites of ztz240 at the voltage-sensing domain (VSD) of KCNQ2. The mutation of F137, one of the identified binding sites, to alanine (A) largely abolished ztz240 activity (Li et al., 2013).

**Figure 1:**
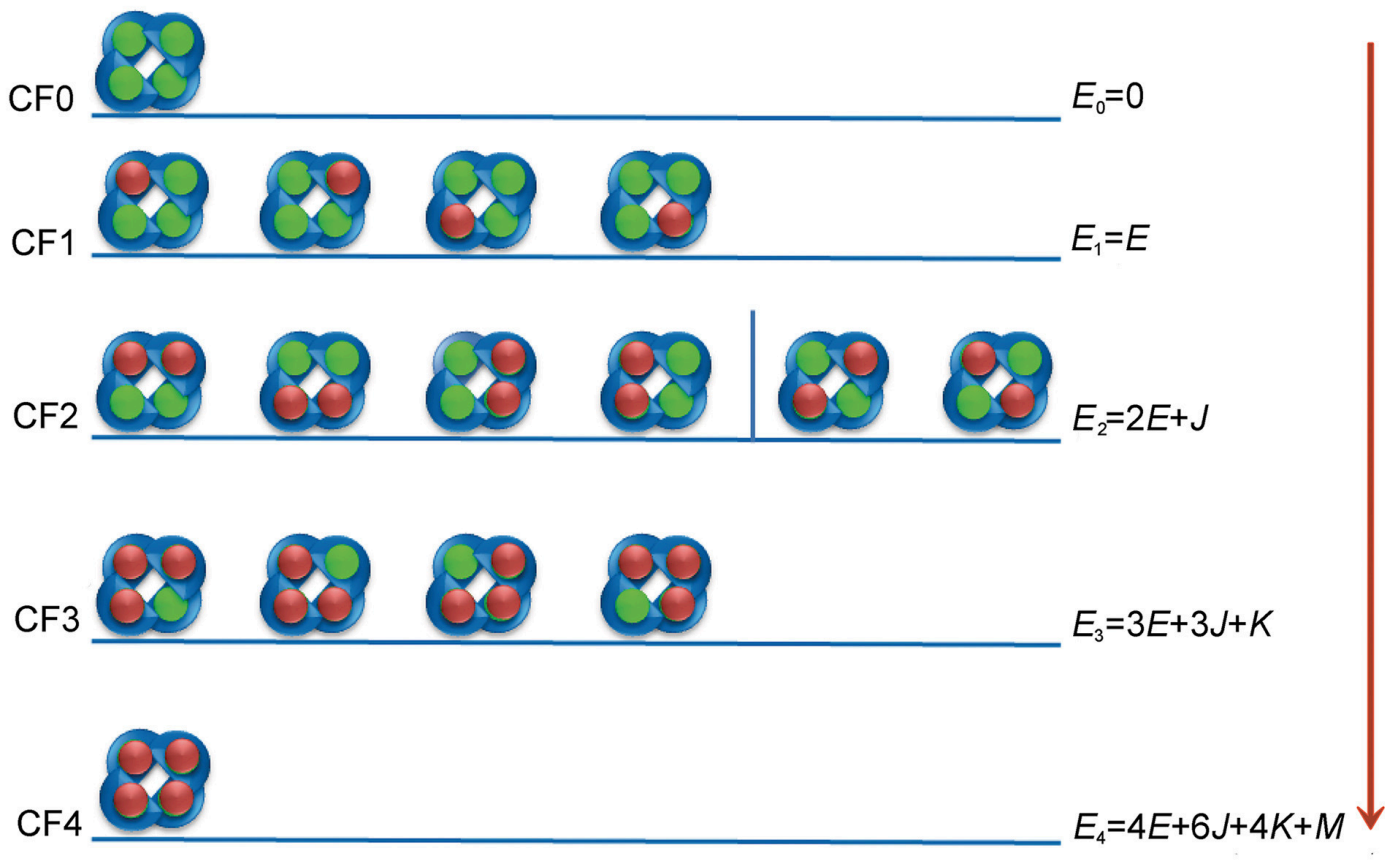
Interaction configurations for ztz240-KCNQ binding. The KCNQ channel is represented by four blue semicircles. The green balls represent non-bonded binding sites on KCNQ, and the red balls indicate the activators.

However, knowledge of the mechanism of interaction of the activators with KCNQ and other potassium ion channels is limited because of the complexity of these mechanisms (Wulff et al., 2009). In general, interactions between activators (or inhibitors) and ion channels are most commonly assessed by measuring the steady state whole-cell current as a function of ligand concentration. Though these concentration-response curves are extensively measured, the data are fitted with an empirical equation referred to as the Hill equation (Hill, 1910; Goutelle et al., 2008). However, the Hill equation does not always reflect the drug action models. As the KCNQ2 channel is composed of four subunits and each subunit has a binding site for activators, upon binding an activator (e.g., ztz240), the channel exhibits five possible configurations, i.e., unliganded, monoliganded, diliganded, triliganded, and tetraliganded forms (hereinafter, we denote these configurations as CF0-CF4, respectively; Figure 1). In principle, upon binding activators, the ion channel undergoes conformational changes that cause the channel to be more open than its intrinsic open state without activator binding, or the activators may stabilize the open conformation of the channel, allowing more potassium ions to flux out the cell (Peretz et al., 2010). Theoretically, the capability for each configuration to transfer potassium ions is different from those of the other configurations. This factor complicates the process of channel activation. Several questions may naturally arise: How do these configurations (CF1-CF4) contribute to the whole activation? Are there any configurations that may dominate the activation process? What is the individual cooperativity of two ligands, three ligands, or four ligands that bind to the channel simultaneously (e.g., cooperativities corresponding to CF2-CF4)? How does one estimate the binding affinity of an activator (or an inhibitor) to an ion channel from the concentration response data? By fitting concentration-response data with the Hill or modified Hill equation, it is impossible to obtain such information. Therefore, a more elaborate mathematical model is required for describing activator-channel binding relations—one that has not previously been developed. To this end, we have established a statistical thermodynamic model for the interaction between activators and potassium ion channels, and we used this model to study the interaction mechanism between ztz240 and the KCNQ2 channel.

The mathematical model presented here assumes that the channel preferentially or cooperatively binds one, two, three, or four activator molecules. Based on this model, an equation for fitting concentration-response data is derived. This quantitative model has the following significance: (i) It provides a good fit to the measured data; the fitting result is much better than fitting by the Hill equation. (ii) It is able to estimate the binding affinity between activator and channel, cooperative coefficients for the binding of multiple activators, and conductance coefficients for CF1-CF4. (iii) It predicts well the concentration-response curves for the heteromeric channel composed of wild-type (WT) subunits and F137A mutant subunits. Remarkably, the prediction is in good agreement with the experimental results determined in parallel with the mathematical modeling.

## Methods

### Statistical thermodynamic model design

#### General description

We use the equilibrium statistical thermodynamic principle, the Boltzmann distribution in particular, and a lattice model (Figure S1) to formulate the mathematical model for the interaction between an activator (or an inhibitor) and an ion channel (a detailed derivation for the model is described in the following section). This paradigm has been widely used in studying complex biological events such as transcription and ligand binding. Typical examples have recently been summarized in several excellent reviews and monographs by Philips et al. (Phillips et al., 2009; Garcia et al., 2010, 2011). This model is also adapted for the interactions between other channels and ligands (activators and inhibitors). The fundamental assumption of the thermodynamic model for a ligand interacting with an iron channel is that we can replace the difficult task of computing the level of channel current potentiation, as measured by the concentration of activator, by solving the more tractable question of the probability (*p*) that the activator occupies the binding sites of the channel. Accordingly, the main focus of the model construction is calculating the probabilities for the different channel configurations that are bound with different numbers of ligand molecules: 1 to 4 for KCNQ2 in the present study.

Our model is developed based on two assumptions. The first key assumption is the idea that the probability of ion channel occupancy by activators is proportional to the electric current passing through the channel. This assumption is consistent with our recent result that ztz240 binds directly to the voltage-sensing domain of KCNQ during activation (Li et al., 2013). The second assumption is that the rates of activators binding to channels are much faster than the time scales for the activators to stimulate the channels. This assumption is in agreement with the general experimental observation that ligand-receptor binding is at the level of a microsecond (Sktar, 1987) and our recent observation that, in the presence of ztz240, the closing of KCNQ2 takes approximately 10 s (Gao et al., 2010). These two assumptions enable us to apply equilibrium thermodynamic tools to develop the activation model of ztz240 binding to KCNQ2.

The validity of our model is ensured by the data reported in this study. We will treat the problem in the canonical ensemble of classical statistical mechanics, with the cell population providing the statistical ensemble of replicas of the system. We will construct the thermodynamic model using the lattice model, a classic physical model that has been widely adopted by Phillips et al. for treating a number of important biological problems, such as transcription, ligand-receptor interactions, phosphorylation of kinases, and ion channel gating (Phillips et al., 2009).

#### Formalism of the interaction model for ligand-channel binding

We assume that the current potentiation efficacy is produced by the occupancy of the activator to the open state of the channel. Although the binding of ztz240 to the closed state cannot be excluded, during the equation derivation, we only consider the binding of the ligand (ztz240 in this study) to the open conformation of a channel (KCNQ2 in this study). In the following, we use ztz240 to indicate a ligand and KCNQ2 to indicate a channel. Figure 1 shows the binding models for ztz240 with KCNQ2, indicating that five configurations (CF0-CF4) should be considered. CF0 is the open conformation of the KCNQ2 channel, and CF1-CF4 represent the open state of KCNQ2 bound by 1–4 ztz240 molecules, respectively. The four subunits of KCNQ2 are arranged as a quadrangle rather than a tetrahedron as indicated by the 3D model (Miceli et al., 2011), and the binding sites of the channel for ztz240 are geometrically located at the four corners of a square (Li et al., 2013). If we treat the configurations as microstates, it is not so difficult to account for the degeneracy (number of states sharing the same energy) of each configuration, i.e., 1 ($C_{4}^{0}$) for CF0, 4 ($C_{4}^{1}$) for CF1, 6 ($C_{4}^{2}$) for CF2, 4 ($C_{4}^{3}$) for CF3, and 1 ($C_{4}^{4}$) for CF4. Of note, CF2 should be divided into two subgroups: the first one includes two ztz240 molecules residing in the two binding sites on one side of the square (the first four structures of CF2), and the second subgroup includes two ztz240 molecules bound to two binding sites on opposing corners of diagonal lines (last two structures of CF2) (Figure 1). In the model derivation of this study, we will not distinguish between these two states.

Next, we set up several parameters.

1. *E*_*s*_, the energy of ztz240 in solution; *E*_0_ = 0, the energy of CF0; *E*_1_ = *E*, the energy of one activator molecule binding to the channel (i.e., the energy of CF1).
2. Cooperativity for two, three, and four ztz240 molecules binding to the channel are captured via additional energies *J, K*, and *M*, respectively.

With these parameters, the energy level of each configuration can be calculated and is shown in Figure 1. These parameters can be transferred to more popularly used constants, such as the binding constant of a ztz240 molecule with one site of KCNQ (*K*_*A*_) and the cooperative coefficients for stabilizing two (γ), three (μ), and four (ν) interacting ztz240 molecules, by Equation (1),

(1) $$K_{A}=e^{-\beta \Delta E},\;\gamma =e^{-\beta J},\;\mu =e^{-\beta K},\;\nu =e^{-\beta M}$$

where Δ*E* = *E*−*E*_*s*_ can be regarded as the binding free energy of one ztz240 molecule to the channel; β = 1/*k*_B_*T*, where *k*_B_ is the Boltzmann constant and *T* is the absolute temperature.

The lattice model is used to deduce the concentration-response relationship of ztz240 activating KCNQ2. The lattice model requires that a large solution box around a channel molecule be divided up into *N* sub-boxes, each of which has molecular dimensions where only one ligand molecule can reside (ztz240 in this study). Figure S1 illustrates two examples for the microstates of the lattice model. Assume that *L* ligands (ztz240 molecules) stray into the solution box around each ion channel (Figure S1). All the ways of arranging *L* ligand molecules among the *N* sub-boxes, i.e., the total number of microstates when no ligands are bound to the channel, can be counted as

(2) $$\Omega_{0}=\frac{N!}{L!\left(N-L\right)!}$$

Now assume that the concentration of ligand is very low and that the box around a channel molecule can be chosen to be as large as possible and can also be divided as many times as possible; thus *N*>>*L*, and Equation (2) can be approximated as,

(3) $$\Omega_{0}\approx \frac{N^{L}}{L!}$$

For each microstate where no ligand is bound to the channel, the total energy is

(4) $$E_{total}^{0}=LE_{s}+E_{0}=LE_{s}$$

In light of these results, the partition function for the system at the state before ligands bind to the channel can be written as,

(5) $$Z_{0}=1\times \frac{N!}{L!\left(N-L\right)!}e^{-\beta LE_{s}}\approx \frac{N^{L}}{L!}e^{-\beta LE_{s}}$$

In a similar way, we can deduce the partition functions for the system at the states of one, two, three, and four ligands binding to the channels, as written in Equations (6–9),

(6) $$Z_{1}=4\times \frac{N!}{\left(L-1\right)!\left(N-L+1\right)!}e^{-\beta \left(L-1\right)E_{s}-\beta E}\approx 4\frac{N^{L-1}}{\left(L-1\right)!}e^{-\beta \left(L-1\right)E_{s}-\beta E}=4\frac{N^{L-1}}{\left(L-1\right)!}e^{-\beta LE_{s}-\beta \Delta E}$$

(7) $$Z_{2}=6\times \frac{N!}{\left(L-2\right)!\left(N-L+2\right)!}e^{-\beta \left(L-2\right)E_{s}-2\beta E-\beta J}\approx 6\frac{N^{L-2}}{\left(L-2\right)!}e^{-\beta \left(L-2\right)E_{s}-2\beta E-\beta J}=6\frac{N^{L-2}}{\left(L-2\right)!}e^{-\beta L-2\beta \Delta E-\beta J}$$

(8) $$Z_{3}=4\times \frac{N!}{\left(L-3\right)!\left(N-L+3\right)!}e^{-\beta \left(L-3\right)E_{s}-3\beta E-3\beta J-\beta K}\approx 4\frac{N^{L-3}}{\left(L-3\right)!}e^{-\beta \left(L-3\right)E_{s}-3\beta E-3\beta J-\beta K}=4\frac{N^{L-3}}{\left(L-3\right)!}e^{-\beta L-3\beta \Delta E-3\beta J-\beta K}$$

(9) $$Z_{4}=1\times \frac{N!}{\left(L-4\right)!\left(N-L+4\right)!}e^{-\beta \left(L-4\right)E_{s}-4\beta E-6\beta J-4\beta K-\beta M}\approx \frac{N^{L-4}}{\left(L-4\right)!}e^{-\beta \left(L-4\right)E_{s}-4\beta E-6\beta J-4\beta K-\beta M}=\frac{N^{L-4}}{\left(L-4\right)!}e^{-\beta L-4\beta \Delta E-6\beta J-4\beta K-\beta M}$$

Factors of 1, 4, 6, 4, and 1 in Equations (5–9) are weighted from the degeneracy. The total partition function of the system considering all states of ligand unbinding and binding is given by,

(10) $$Z=Z_{0}+Z_{1}+Z_{2}+Z_{3}+Z_{4}$$

Accordingly, the probabilities of a KCNQ2 channel being occupied by zero, one, two, three, and four ligands, i.e., probabilities for CF0-CF4 in Figure 1, can be written as

(11) $$p_{0}=\frac{Z_{0}}{Z},p_{1}=\frac{Z_{1}}{Z},p_{2}=\frac{Z_{2}}{Z},p_{3}=\frac{Z_{3}}{Z},p_{4}=\frac{Z_{4}}{Z}$$

Substituting Equations (5–9) into Equation (10), the probabilities for CF0-CF4 become

(12) $$p_{0}=\frac{1}{1+4\frac{L}{N}e^{-\beta \Delta E}+6{\left(\frac{L}{N}\right)}^{2}e^{-2\beta \Delta E-\beta J}+4{\left(\frac{L}{N}\right)}^{3}e^{-3\beta \Delta E-3\beta J-\beta K}+{\left(\frac{L}{N}\right)}^{4}e^{-4\beta \Delta E-6\beta J-4\beta K-\beta M}}$$

(13) $$p_{1}=\frac{4\frac{L}{N}e^{-\beta \Delta E}}{1+4\frac{L}{N}e^{-\beta \Delta E}+6{\left(\frac{L}{N}\right)}^{2}e^{-2\beta \Delta E-\beta J}+4{\left(\frac{L}{N}\right)}^{3}e^{-3\beta \Delta E-3\beta J-\beta K}+{\left(\frac{L}{N}\right)}^{4}e^{-4\beta \Delta E-6\beta J-4\beta K-\beta M}}$$

(14) $$p_{2}=\frac{6{\left(\frac{L}{N}\right)}^{2}e^{-2\beta \Delta E-\beta J}}{1+4\frac{L}{N}e^{-\beta \Delta E}+6{\left(\frac{L}{N}\right)}^{2}e^{-2\beta \Delta E-\beta J}+4{\left(\frac{L}{N}\right)}^{3}e^{-3\beta \Delta E-3\beta J-\beta K}+{\left(\frac{L}{N}\right)}^{4}e^{-4\beta \Delta E-6\beta J-4\beta K-\beta M}}$$

(15) $$p_{3}=\frac{4{\left(\frac{L}{N}\right)}^{3}e^{-3\beta \Delta E-3\beta J-\beta K}}{1+4\frac{L}{N}e^{-\beta \Delta E}+6{\left(\frac{L}{N}\right)}^{2}e^{-2\beta \Delta E-\beta J}+4{\left(\frac{L}{N}\right)}^{3}e^{-3\beta \Delta E-3\beta J-\beta K}+{\left(\frac{L}{N}\right)}^{4}e^{-4\beta \Delta E-6\beta J-4\beta K-\beta M}}$$

(16) $$p_{4}=\frac{{\left(\frac{L}{N}\right)}^{4}e^{-4\beta \Delta E-6\beta J-4\beta K-\beta M}}{1+4\frac{L}{N}e^{-\beta \Delta E}+6{\left(\frac{L}{N}\right)}^{2}e^{-2\beta \Delta E-\beta J}+4{\left(\frac{L}{N}\right)}^{3}e^{-3\beta \Delta E-3\beta J-\beta K}+{\left(\frac{L}{N}\right)}^{4}e^{-4\beta \Delta E-6\beta J-4\beta K-\beta M}}$$

If we assume the overall volume of the large box around a channel is V, then

(17) $$\frac{L}{N}=\frac{\frac{L}{V}}{\frac{N}{V}}=\frac{\left[L\right]}{\left[L_{0}\right]}$$

Here, [*L*] is the concentration of the activator, and [*L*_0_] is a “reference” concentration where every lattice sub-box is occupied. The choice of reference concentration is arbitrary. For the sake of convenience, we can choose the sub-box size to be the appropriate value, resulting in [*L*_0_] = 1 M. Meanwhile, substituting the binding constant and cooperative coefficients described in Equation (1) into Equations (12–16), the probabilities are given by

(18) $$p_{0}=\frac{1}{1+4\left[L\right]K_{A}+6{\left[L\right]}^{2}K_{A}^{2}\gamma +4{\left[L\right]}^{3}K_{A}^{3}\gamma^{3}\mu +{\left[L\right]}^{4}K_{A}^{4}\gamma^{6}\mu^{4}\nu }$$

(19) $$p_{1}=\frac{4\left[L\right]K_{A}}{1+4\left[L\right]K_{A}+6{\left[L\right]}^{2}K_{A}^{2}\gamma +4{\left[L\right]}^{3}K_{A}^{3}\gamma^{3}\mu +{\left[L\right]}^{4}K_{A}^{4}\gamma^{6}\mu^{4}\nu }$$

(20) $$p_{2}=\frac{6{\left[L\right]}^{2}K_{A}^{2}\gamma }{1+4\left[L\right]K_{A}+6{\left[L\right]}^{2}K_{A}^{2}\gamma +4{\left[L\right]}^{3}K_{A}^{3}\gamma^{3}\mu +{\left[L\right]}^{4}K_{A}^{4}\gamma^{6}\mu^{4}\nu }$$

(21) $$p_{3}=\frac{4{\left[L\right]}^{3}K_{A}^{3}\gamma^{3}\mu }{1+4\left[L\right]K_{A}+6{\left[L\right]}^{2}K_{A}^{2}\gamma +4{\left[L\right]}^{3}K_{A}^{3}\gamma^{3}\mu +{\left[L\right]}^{4}K_{A}^{4}\gamma^{6}\mu^{4}\nu }$$

(22) $$p_{4}=\frac{{\left[L\right]}^{4}K_{A}^{4}\gamma^{6}\mu^{4}\nu }{1+4\left[L\right]K_{A}+6{\left[L\right]}^{2}K_{A}^{2}\gamma +4{\left[L\right]}^{3}K_{A}^{3}\gamma^{3}\mu +{\left[L\right]}^{4}K_{A}^{4}\gamma^{6}\mu^{4}\nu }$$

The contribution of each configuration to the whole cell current should be proportional to its probability. Considering that the conductance of all configurations are different from each other, four new parameters, *a, b, c*, and *d*, are defined to refer to the capabilities of flowing current for CF1 to CF4, respectively. Therefore, the normalized current potentiated by the activator can be written as,

(23) $$\Delta I/\Delta I_{\max}\;=ap_{1}+bp_{2}+cp_{3}+dp_{4}$$

Substituting Equations (18–22) into Equation (23), we obtain a concentration-response equation, which provides a statistical thermodynamic translation of the model assumptions into electrophysiologically significant quantities and can be used to fit the experimental data measured by recording the steady state whole cell current as a function of activator concentration.

Thus far, Equation (23) is expressed in terms of eight parameters, {*K*_*A*_, γ, μ, ν; *a, b, c, d*}. By fitting the concentration-response data of an activator potentiating a channel, we obtain these parameters. These parameters provide insight into the binding affinity of the activator with the channel; the cooperative effects of two, three, and four activator molecules binding to the channel together; and the channel conductance properties of each configuration (CF1-CF4). Substituting the values of these parameters into Equations (18–22), we can obtain the occupancy probability of each configuration and further deduce a possible action mechanism of the activator activating the channel.

### Cell culture and electrophysiological assays

Chinese hamster ovary (CHO) cells were grown in 50/50 DMEM/F12 (Cellgro, Manassas, VA) with 10% fetal bovine serum (FBS), and 2 mM L-glutamine (Gibco, Carlsbad, CA). To express ion channels, cells were split 24 h before transfection, plated in 60-mm dishes, and transfected with Lipofectamine 2000™ reagent (Invitrogen, Carlsbad, CA) according to the manufacturer's instruction. At 24 h after transfection, the cells were split and re-plated onto coverslips coated with poly-L-lysine (Sigma-Aldrich, St. Louis, MO). A plasmid for cDNA of GFP (Amaxa, Gaithersburg, MD) was cotransfected to aid identification of transfected cells by fluorescence microscopy. To co-express wild-type and mutant KCNQ2, the cDNA concentration of wild-type KCNQ2 and the mutant was adjusted to 200 ng/μl, and the molar ratio was 1:1.

Whole-cell voltage clamp recording was carried out using cultured CHO cells at room temperature with an Axopatch-200B amplifier (Molecular Devices, Sunnyvale, CA). The electrodes were pulled from borosilicate glass capillaries (World Precision Instruments, Sarasota, Fl). When filled with the intracellular solution, the electrodes had resistances of 3–5 MΩ. The pipette solution contained the following (mM): KCl 145, MgCl_2_ 1, EGTA 5, HEPES 10, MgATP 5 (pH = 7.3 with KOH). During the recording, constant perfusion of extracellular solution was maintained using a BPS perfusion system (ALA Scientific Instruments, Westburg, NY). The extracellular solution contained the following (mM): NaCl 140, KCl 3, CaCl_2_ 2, MgCl_2_ 1.5, HEPES 10, and glucose 10 (pH = 7.4 with NaOH). Signals were filtered at 1 KHz and digitized using a DigiData 1440 with pClamp 9.2 software (Molecular Devices, Sunnyvale, CA). Series resistance was compensated by 60–80%. In the present study, the holding potential was −120 mV in all voltage protocols, except where indicated. To elicit the currents, cells were stimulated by depolarization steps as indicated. All data points represent the mean ± SEM.

### Validation and application of the theoretical model

To validate our model, we fitted it to the concentration-response data of ztz240 to the KCNQ2 channel by using the curve fitting toolbox “cftool” encoded in MatLab software (Liu et al., 2006). We fitted the normalized experimental data (Δ*I/*Δ*I*_max_ values) for WT KCNQ2 using Equation (23). Without any previous knowledge of the parameters, we set estimates randomly and used them as initial conditions in a Trust-Region nonlinear-least square fitting procedure to refine the solution. To reduce or even avoid initial estimate dependence, we repeated our fitting procedure with a series of different random initial estimates and obtained the most reliable and stable solution as our ultimate solution to the parameters of the model. Two main statistical measures generated by the cftool were used to determine the best of fit: the sum of squares due to error (SSE) and the R-square statistics (R-square) (value ϵ [0, 1]) between the experimental and calculated values for models (definition of equations in SI text). A value of SSE closer to 0 indicates that the model has a smaller random error component, and that the fit will be more useful for prediction. For R-square, a value closer to 1 indicates that a greater proportion of variance is accounted for by the model. Therefore, higher R-square close to 1 with lower SSE close to 0 will indicate a better curve fitting. We randomly set the initial values for several rounds of fitting and determine the best fitting based on the two above mentioned measures, that is, the fitting which has the minimum SSE and maximum R-square. Actually, the fittings were relatively stable, and the results of the determined parameters of the models were very similar. The default fit convergence criteria were used: maximum number of function evaluations allowed was set as 600; maximum number of fit iterations allowed was 400; termination tolerance used on stopping conditions involving the function value was 10^−6^; and termination tolerance used on stopping conditions involving the coefficients was also 10^−6^. Moreover, a further analysis on the mathematical foundation of the model fitting results is given in SI text.

**Table 1:** Experimental concentration-response data (***I***/***I***_0_) and corresponding normalized data (Δ***I***/Δ***I***_max_) of ztz240 potentiating WT KCNQ2 and WT-F137A heteromeric KCNQ2.

**Log[ztz240]**	**WT channel**		**WT–F137A heteromeric channel**		
	**Exp. *I/I*_0_**	**Exp. Δ*I/*Δ*I*_max_**	**Exp. *I/I*_0_**	**Exp. Δ*I/*Δ*I*_max_**	**Pred. WT Δ*I/*Δ*I*_max_**
−8.0	1.03, 0.03	0.0210, 0.02	1.02, 0.01	0.0150, 0.008	0.00866
−7.0	1.09, 0.09	0.0636, 0.06	1.12, 0.08	0.0902, 0.06	0.0750
−6.0	1.20, 0.09	0.139, 0.06	1.29, 0.07	0.218, 0.05	0.231
−5.5	1.35, 0.2	0.245, 0.2	1.13, 0.02	0.0977, 0.02	0.275
−5.0	1.90, 0.4	0.627, 0.3	1.48, 0.07	0.361, 0.05	0.359
−4.0	2.43, 0.5	1.00, 0.3	2.12, 0.1	0.842, 0.09	0.911
−3.5	–^*^	–^*^	2.33, 0.3	1.00, 0.2	0.981

*I/I_0_ indicates the effects of ztz240 on the amplitude of the outward current of KCNQ channels. I_0_ is the amplitude of the outward current in the absence of the compound; I is the amplitude of the outward current in the presence of the compound; ΔI/ΔI_max_ is the normalized change in the currents before and after ztz240. The holding potential was −120 mV. To elicit the outward currents, the cells were depolarized to −10 mV. Data are presented as the means ± SE (n = 8–10)*.

* Data cannot be measured

## Results

### Experimental data fitting to the statistical thermodynamic model

Having established the statistical thermodynamic model, we used it to fit the experimental data. Before that, we obtained a set of concentration-response data for ztz240 potentiating KCNQ2 by determining the outward current (*I/I*_0_ values) at different concentrations of ztz240 (Table 1). To fit experimental data by Equation (23), *I/I*_0_ values were normalized to Δ*I/*Δ*I*_max_ values, which are also listed in Table 1. The best-fit parameters are as follows,

(24) $$\begin{array}{l} \left\{K_{A},\gamma ,\mu ,\nu ;a,b,c,d\right\}\\ =\left\{\begin{array}{l} 1.805\times 10^{5}M^{-1},0.928M^{-1},0.4529M^{-1},65.24M^{-1};\\ 0.3083,0.2375,0.4011,0.9863 \end{array}\right\} \end{array}$$

**Figure 2:**
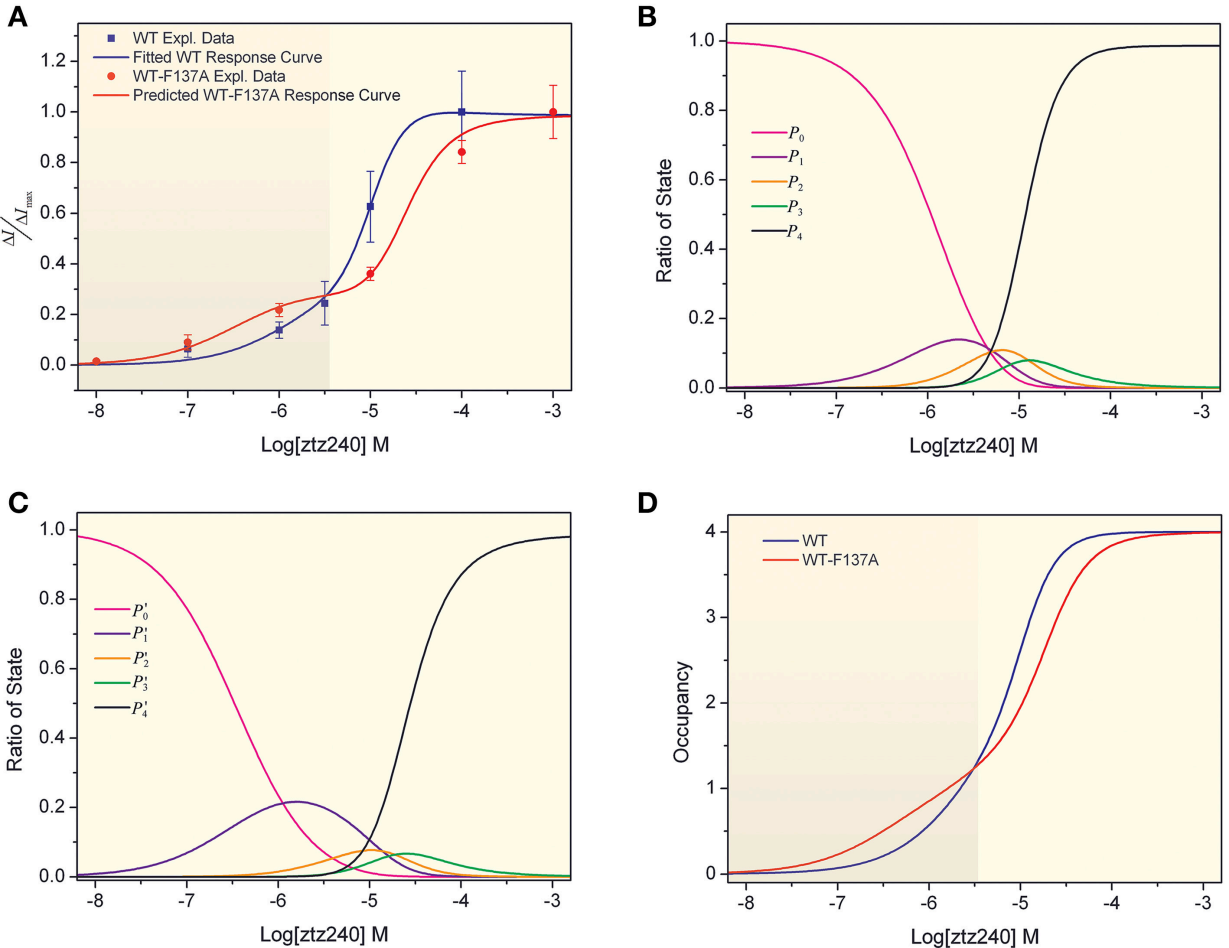
Concentration-response curves of ztz240 to WT channels and heteromeric K^+^ channels. **(A)** Concentration-response curve of ztz240 to WT channels fitted by our presented model and the concentration-response curve of ztz240 to heteromeric channels predicted by our model; **(B)** the probabilities of finding zero, one, two, three, and four ztz240 molecules bound to WT KCNQ2 (*P*_0_–*P*_4_) as functions of the logarithms of ztz240 concentrations (Log[ztz240]); **(C)** the predicted probabilities of zero, one, two, three, and four ztz240 molecules bound to WT-F137A KCNQ2 (*P*_0_–*P*_4_) as functions of the logarithms of ztz240 concentrations (Log[ztz240]); and **(D)** the concentration-response curves of average numbers of bound ztz240 molecules on both WT and WT-F137A heteromeric channels.

With the best-fit parameters, we obtained a concentration-response curve for ztz240 activating KCNQ2, which fit the experimental data well (Figure 2A). To validate the goodness of fit of our model to the experimental data, we calculated the sum of squares due to error (SSE) and the *R*-square (*R*^2^) between the experimental response values and predicted values. The value of SSE (0.002468) is close to 0, indicating that our model has a smaller random error component; the value of *R*^2^ (0.9974) is closer to 1, indicating that a greater proportion of the variance is accounted for by our model. According to our model, the dissociation constant (*K*_D_ = 1/*K*_*A*_) of one ztz240 molecule unbinding from KCNQ2 can be estimated to be ~5.54 μM, and the EC_50_ value (the ztz240 concentration at which Δ*I/*Δ*I*_max_ is 50% of its maximum value) is predicted to be ~7.41 μM, which is quite close to the obtained experimental value, ~5.80 μM. These results indirectly indicate the reliability of our model and data fitting procedure. The reliability of our model will be demonstrated further by its capability of predicting the concentration-response curve for ztz240 potentiating the heteromeric channel (see results below).

The parameters from the data fitting by our theoretical model suggest that two ztz240 molecules binding together to the channel would not show any cooperative effect, as indicated by γ = 0.9280 M^−1^, and three ztz240 molecules binding to the channel might have a weak negative cooperative effect due to μ = 0.4529 M^−1^. However, the cooperativity for four molecules of ztz240 binding simultaneously to the channel is notable; the coupling energy is 2.47 kcal/mol as estimated for the cooperative coefficient, ν = 65.24 M^−1^ (Equation 24).

By substituting the values of the eight parameters listed in Equation (24) into Equations (18–22), we can compute the probabilities of the allowed configurations for ztz240 binding to KCNQ2 (CF0-CF4) at different concentrations. The result is shown in Figure 2B, which plots the probabilities of finding zero, one, two, three, and four ztz240 molecules bound to KCNQ2 (*p*_0_-*p*_4_, respectively) as functions of the logarithms of ztz240 concentrations (Log[ztz240]). As expected, the plot of *p*_0_ monotonously decreased due to more channel molecules being occupied by ztz240 molecules as the ztz240 concentration increased. The plots of *p*_1_–p_3_ are normally distributed as bell curves, and the concentrations of ztz240 corresponding to the peaks of the curves are in the order of *p*_1_ < *p*_2_ < *p*_3_. At low concentrations, configurations with one or two ztz240 molecules (CF1 and CF2) are the dominant binding states. Owing to a strong cooperative effect, the four ztz240 molecules occupied configuration (CF4) is the dominant binding state at relatively high concentrations of ztz240. Due to the negative cooperativity of three ztz240 molecules binding to the channel, the occupying probability of CF3 is not as significant as expected.

### Extension of the model to WT-F137A heteromeric channel system

The above model fits the concentration-response data of ztz240 activating KCNQ2 channel well, and the result is much better than fitting with the Hill equation (see results below). To further verify the reliability of the model and the accuracy of the fitted parameters, we tried to predict the concentration-response curve for the heteromeric channel of wild-type subunits of KCNQ2 with subunits containing F137A mutant (hereinafter, we designate it as the heteromeric channel). Co-expression of an equal molar ratio of WT and F137A mutant channel may generate a mixture with various stoichiometries of heteromeric channels. Note that combination of the two kinds of subunits may form five tetramers as indicated in Figure S2, i.e., (KCNQ2)_4_ (pure wild-type (WT) channel), (KCNQ2)_3_•(F137A)_1_, (KCNQ2)_2_•(F137A)_2_, (KCNQ2)_1_•(F137A)_3_, and (F137A)_4_ (pure F137A mutant channel). We can account for the microstate multiplicities (combinations between wild-type subunits and mutant subunits) for these five tetramer channels, which are 1, 4, 6, 4, and 1, respectively (Figure S2). Our experimental determination showed that ztz240 almost lost the ability to potentiate the pure F137A mutant channel, implying the activator is not able to bind to the mutated subunits at the applied concentrations (Li et al., 2013). Additionally, the mutant channel maintains the general electrophysiological function of the wild-type channel (Figure S3). Considering these observations, we can assume that the F137A mutation might not seriously influence the interaction and cooperative effects of ztz240 on the wild-type subunit and that the channel conductance for each configuration of the mutant channel is almost as same as that of the WT channel. Based on this assumption and the experimental results, we can calculate the microstate multiplicities for CF0-CF4 of the heteromeric channel, which are 2, 32, 24, 8 and 1, respectively. Figure S4 shows an example of how to calculate the multiplicity for CF2. The occupancy probabilities of the configurations with zero, one, two, three, and four ztz240 molecules bound to the heteromeric channels (i.e., CF0-CF4) can be computed with Equations (25–29), respectively (for a detailed description of the deviation see SI text).

(25) $$p\prime_{0}=\frac{2}{2+32\left[L\right]K_{A}+24{\left[L\right]}^{2}K_{A}^{2}\gamma +8{\left[L\right]}^{3}K_{A}^{3}\gamma^{3}\mu +{\left[L\right]}^{4}K_{A}^{4}\gamma^{6}\mu^{4}\nu }$$

(26) $$p\prime_{1}=\frac{32\left[L\right]K_{A}}{2+32\left[L\right]K_{A}+24{\left[L\right]}^{2}K_{A}^{2}\gamma +8{\left[L\right]}^{3}K_{A}^{3}\gamma^{3}\mu +{\left[L\right]}^{4}K_{A}^{4}\gamma^{6}\mu^{4}\nu }$$

(27) $$p\prime_{2}=\frac{24{\left[L\right]}^{2}K_{A}^{2}\gamma }{2+32\left[L\right]K_{A}+24{\left[L\right]}^{2}K_{A}^{2}\gamma +8{\left[L\right]}^{3}K_{A}^{3}\gamma^{3}\mu +{\left[L\right]}^{4}K_{A}^{4}\gamma^{6}\mu^{4}\nu }$$

(28) $$p\prime_{3}=\frac{8{\left[L\right]}^{3}K_{A}^{3}\gamma^{3}\mu }{2+32\left[L\right]K_{A}+24{\left[L\right]}^{2}K_{A}^{2}\gamma +8{\left[L\right]}^{3}K_{A}^{3}\gamma^{3}\mu +{\left[L\right]}^{4}K_{A}^{4}\gamma^{6}\mu^{4}\nu }$$

(29) $$p\prime_{4}=\frac{{\left[L\right]}^{4}K_{A}^{4}\gamma^{6}\mu^{4}\nu }{2+32\left[L\right]K_{A}+24{\left[L\right]}^{2}K_{A}^{2}\gamma +8{\left[L\right]}^{3}K_{A}^{3}\gamma^{3}\mu +{\left[L\right]}^{4}K_{A}^{4}\gamma^{6}\mu^{4}\nu }$$

The normalized current potentiation of the heteromeric channels activated by ztz240 can be written as

(30) $$\Delta I^{\prime }/\Delta I_{\max}^{\prime }=ap\prime_{1}+bp\prime_{2}+cp\prime_{3}+dp\prime_{4}$$

This equation can be used to predict the concentration-response curve for the heteromeric channel. Additionally, Equation (30) can be used to fit the experimental data or the heteromeric channel measured by recording the steady state whole-cell current as a function of ztz240 concentration.

### Prediction of the concentration-response curve for the WT-F137A heteromeric channel and agreement with experimental data

In fact, experimentally measuring the parameters {γ, μ, ν; a, b, c, d} is very difficulty. Considering the challenges in the purification of membrane proteins and the relatively low binding affinity of channel activators, it is also difficult to determine the binding constant between an activator and an ion channel (e.g., *K*_*A*_) experimentally. However, we can obtain these parameters by fitting our mathematical mode to the experimental concentration-response data. Then we can predict the concentration-response curve for the binding of other ligands based on our parameterized model. Therefore, to further verify the reliability of our model, we predicted a concentration-response curve for ztz240 potentiating the WT-F137A heteromeric channels by substituting the above-obtained values of the eight parameters into Equations (25–30). The predicted concentration-response curve is also shown in Figure 2A. In parallel, we experimentally determined the concentration-response data of ztz240 to the heteromeric channel (see SI text for details). Surprisingly, the experimental concentration-response data are very close to the predicted data (Table 1), with the SSE value between the predicted data and experimental data for the six concentrations of ztz240 being only 0.0056, and with an *R*^2^ as high as 0.9933. Pictorially, the experimental points closely cling to the predicted curve (Figure 2A). Consistency between the prediction and the additional experiment again verified the reliability of our statistical thermodynamic model for activators to potentiate K^+^ channels.

Intuitively, the whole concentration-response curve of a drug will fully shift to the right or left in response to changes in the drug binding receptor (Schenzer et al., 2005; Wang et al., 2005; Xing et al., 2010). However, the concentration-response curve of ztz240 binding to the heteromeric channel is not a normal sigmoid plot as a function of Log[ztz240]. A striking feature of the curve is that it shifts toward the left at a lower concentration of ztz240 (Log[ztz240] < −5.4) and shifts considerably to the right at a higher concentration of ztz240 (Log[ztz240] > −5.4). Thus, the curve is composed of two sigmoid curves (Figure 2A) (hereinafter, we designate it as a bi-sigmoid curve). This bi-sigmoid curve reveals that, at lower concentrations, ztz240 is more sensitive to the heteromeric channel than to the WT channel, and at higher concentrations, ztz240 is inclined to the WT channel. This result might provide new insight for studying the drug action mechanisms of the heteromeric channels composed of WT subunits and hereditary mutants (Schroeder et al., 1998). In addition, in comparison with WT KCNQ2, the plot of *p*′_1_ expands outward, those of *p*′_2_ and *p*′_3_ squash downward, and that of *p*′_4_ shifts toward a higher ztz240 concentration (Figure 2C). This result indicates that the contribution of one molecule binding configuration (CF1) increases and those of other configurations (CF1-CF4) decrease during ztz240 activating the heteromeric channel. Thus, the probability plots of the activator bound configurations give a mathematical explanation for the bi-sigmoid curve.

### Advantage of our statistical thermodynamic model over the hill equation

**Figure 3:**
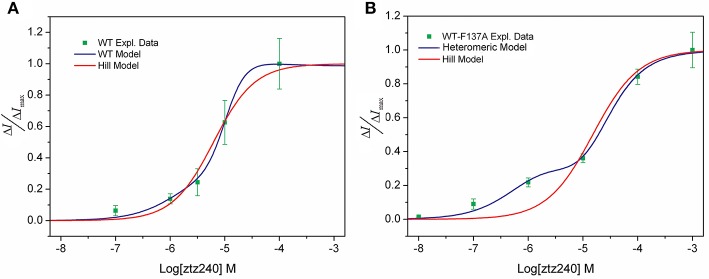
Comparison of our model (blue line) and the Hill model (red line) of the concentration-response fit to the experimental observed data (green squares). **(A)** WT KCNQ2 model and **(B)** heteromeric KCNQ2 model.

We tested the concentration-response data of both WT and WT-F137A heteromeric channels to fit the widely applied Hill equation. Our unfavorable results indicate that the Hill equation is not able to fit well to our experimental data. For the WT channel, the Hill equation produced a relatively larger SSE (0.01803) and a smaller *R*^2^ (0.9790; Figure 3A). For the heteromeric channel, the Hill equation loses the capability of fitting the concentration-response data, giving a sigmoid curve rather than a bi-sigmoid curve. This fitting result is not in agreement with the experimental result (Figure 3B). Mathematically, the Hill equation cannot give a bi-sigmoid curve because the Δ*I/*Δ*I*_max_-Log[*L*] plot described by this equation contains only one inflection point at [*L*] = *EC*_50_ (see SI text). On the other hand, both the numerator and denominator of either Equation (23) or Equation (30) are polynomial functions of [*L*]; thereby, the Δ*I/*Δ*I*_max_-Log[*L*] plot described by these two equations could have multiple inflection points (see SI text). This is the mathematical foundation that shows our statistical thermodynamic model can fit the experimental data well.

### Implication from average occupancies

With the fitted parameters, we can calculate the partition functions (*Z*) for ztz240 binding to both the WT KCNQ2 channel and WT-F137A heteromeric channel. Thus, we can find the average occupancy (<*N*_*bound*_>) by evaluating

(31) $$\langle N_{bound}\rangle =\frac{1}{\beta }\left(\frac{\partial \ln Z}{\partial \Delta E}\right)=\sum_{i=1}^{4}ip_{i}$$

where Δ*E* is the binding free energy of one ztz240 molecule to the channel; β = 1/*k*_B_*T*, where *k*_B_ is the Boltzmann constant, *T* is absolute temperature, and *p*_i_ refers to the probability of the configuration with *i* ztz240 molecules bound to the channel. Using this paradigm, we calculated the concentration dependences of average numbers of bound ztz240 molecules on both WT and WT-F137A heteromeric channels (Figure 2D). The occupancy curve of ztz240 to the WT channel is steeper than that of the heteromeric channel, implying that the whole cooperativity of ztz240 to the WT channel is stronger than that of the heteromeric channel.

## Discussion and conclusions

Various theoretical models have been developed to analyze ligand-protein interactions. However, none of them address the problem of activators potentiating ion channels. Based on the statistical thermodynamic principles, we have deduced a quantitative model for the interactions between activators and K^+^ channels. Although we have focused on KCNQ2 in the present study, our model is also applicable to other members of the superfamily of voltage-gated 6-transmembrane K^+^ channels. By fitting experimental concentration-response data, our model gives eight parameters, {*K*_*A*_, γ, μ, ν; *a, b, c, d*}. These parameters provide a general picture of the activation mechanism and the binding affinity of an activator with an iron channel; the cooperative effects of one, two, three and four activator molecules binding to the channel together; and the channel conductance properties of each binding configuration (CF1-CF4) (i.e., *a–d* in Equations 23 and 30).

In the present study, we have demonstrated that our model provides an excellent fit to the concentration-response data of ztz240 activating the KCNQ2 channel (Figure 2A); the fitting result is much better than the fit by the widely used Hill equation (Figure 3). Note that whole-cell currents, not the single-channel currents, were measured for validation in our study. We found that, without any adjustment of parameters produced by fitting the concentration-response data of ztz240 potentiating the WT KCNQ2 channel, the concentration-response curve of ztz240 activating the WT-F137A heteromeric channel predicted by our statistical thermodynamic model is in good agreement with the experimental data determined here in parallel. This lends credence to the assumptions on which the model is based and to the model itself.

This study yielded an unexpected finding that the concentration-response curve of ztz240 activating the WT-F137A heteromeric channel is bi-sigmoidal, shifting toward the left at lower concentrations of ztz240 and toward the right at higher concentrations of ztz240 (Figure 2A). This situation is infrequent among the reported concentration-response curves because the concentration-response curve of a drug was assumed to be fully left-shifted or right-shifted in response to the changes of the drug binding receptor. The Hill equation completely failed to fit such concentration-response data (Figure 3B). Surprisingly, our model naturally predicted such a bi-sigmoid curve for the WT-F137A heteromeric channel, which fits the experimental data well (Figure 2A). This unexpected result, from another standpoint, reflects the reliability of our model.

## Author contributions

FB, ZG, and HJ: Designed the research and wrote the paper; FB, XP, PL, PZ, HY, XW, ML, ZG, and HJ: Performed the research; FB, PX, PL, ML, ZG, and HJ: Analyzed the data. All authors approved the final version of the manuscript.

### Conflict of interest statement

The authors declare that the research was conducted in the absence of any commercial or financial relationships that could be construed as a potential conflict of interest. The reviewer ZZ declared a shared affiliation, with no collaboration, with several of the authors, ZG and HJ, to the handling Editor.

## Abbreviations

KCNQ2: potassium channel, voltage gated KQT-like subfamily Q, member 2
VSD: voltage-sensing domain
WT: wild-type
SSE: sum of squares due to error.
