# Supplementary material for: A Statistical Thermodynamic Model for Ligands Interacting With Ion Channels: Theoretical Model and Experimental Validation of the KCNQ2 Channel
Source: Front Pharmacol. 2018 Mar 9;9:150. doi: 10.3389/fphar.2018.00150 (PMC5855359; doi:10.3389/fphar.2018.00150)
Supplement: Supplementary file 1 [file DataSheet1.pdf]

Supporting Information

A statistical thermodynamic model for ligands interacting with ion channels: theoretical model and experimental validation of the KCNQ2 channel

Short running title: A thermodynamic model for ligand-ion channel interactions

Fang Bai[1,2,†,‡], Xiaoping Pi[2,3,†], Ping Li[2], Pingzheng Zhou[2], Huaiyu Yang[2], Xicheng Wang[1], Zhaobing Gao[2,3*‡] and Hualiang Jiang[2,3*‡]

[1]Department of Engineering Mechanics, State Key Laboratory of Structural Analysis for Industrial Equipment, and Faculty of Chemical, Environmental, and Biological Science and Technology, Dalian University of Technology, Dalian 116023, China; [2]Drug Discovery and Design Center, State Key Laboratory of Drug Research, CAS Key Laboratory of Receptor Research, Shanghai Institute of Materia Medica, Chinese Academy of Sciences, Shanghai 201203, China; [3]University of Chinese Academy of Sciences, No. 19A Yuquan Road, Beijing 100049, China.

Correspondence: Prof. Hualiang Jiang or Prof. Zhaobing Gao, Drug Discovery and Design Center, State Key Laboratory of Drug Research, Shanghai Institute of Materia Medica, Chinese Academy of Sciences, Shanghai 201203, China. Email: hljiang@simm.ac.cn or zbgao@simm.ac.cn.

[†]These authors contributed equally.

[*]These authors contributed equally as senior authors.

[‡]Current Address: Center for Theoretical Biological Physics, Rice University, TX 77005, United States.

Formalism of the interaction model for interaction between ztz240 and the heterozygous channel of wild-type KCNQ2 with F137A mutant

Recently, we have identified the binding sites for ztz240 in KCNQ2. There are four binding pockets at the whole channel, each subunit contains one. F137 is a key residue lining the binding pocket for each subunit. ztz240 almost is not capable of potentiating the current of F137A mutant channel, implying that the activator is almost not able to bind to the mutated pockets.(Li *et al.*, 2013) This result gives us an opportunity to design experiment to verify the reliability of our statistical thermodynamic model.

We applied ztz240 to the wild-type and F137A mutant (1:1) heterozygous channel. Eqs. 17-22 (main text) cannot be applied to fit the data of the heterozygous channel. Accordingly, we deduced formulae for fitting the experimental data of the heterozygous channel. Because mutated subunit are added into the cellular membrane, four new molecular species of the KCNQ2 should be existed, which are $(KCNQ2)_3$•$(F137A)_1$, $(KCNQ2)_2$•$(F137A)_2$, $(KCNQ2)_1$•$(F137A)_3$, and $(F137A)_4$. The schematic pictures and corresponding multiplicities for all the five molecular species are shown in Figure S2. We assume that, on the WT and mutant subunits heterozygous channels, only the WT subunits contain binding sites for ztz240 (Figure S2). Another assumption is that F137A mutations at one, two or three units do not affect the binding of ztz240 to the rest wild-type subunit(s), i.e. K_A values of ztz240 to the mixture channels are as the same as that of ztz240 to the WT channel.

Because the WT and mutated subunits can form five channels ($(KCNQ2)_3$ (i.e. $(KCNQ2)_4$, $(KCNQ2)_2$•$(F137A)_2$, $(KCNQ2)_1$•$(F137A)_3$, and $(F137A)_4$) and each channel has multiple microstates (Figure S2), the way to calculate the degeneracy for each activator binding configuration of the heterozygous channel is slightly more complicated than that of WT channel. Electrophysiological test indicated that the mutation F137A does not change the electrophysiological property of the channel (Figure S3). Therefore, $(F137A)_4$ and $(KCNQ2)_4$ can be categorized into the CF0 configuration. The degeneracy of CF0 is 2 rather than 1. The degeneracy for each of other binding configurations can be respectively given by

CF1: $C_4^1 + 4\times C_3^1 + 6\times C_2^1 + 4 = 32$;

CF2: $C_4^2 + 4\times C_3^2 + 6\times C_2^2 = 24$;

CF3: $C_4^3 + 4\times C_3^3 = 8$;

CF4: $C_4^4=1$.

Figure S4 gives an example for the microstates describing that two activators interact with the mixture channel. In a similar way to deduce Eqs. 18-22 (main text), the probabilities for CF0-CF4 of the heterozygous channel can be written as

$$p'_0=\frac{2}{2+32[L]K_A+24[L]^2K_A^2\gamma+8[L]^3K_A^3\gamma^3\mu+[L]^4K_A^4\gamma^6\mu^4\nu}\qquad[1]$$

$$p'_1=\frac{32[L]K_A}{2+32[L]K_A+24[L]^2K_A^2\gamma+8[L]^3K_A^3\gamma^3\mu+[L]^4K_A^4\gamma^6\mu^4\nu}\qquad[2]$$

$$p'_2=\frac{24[L]^2K_A^2\gamma}{2+32[L]K_A+24[L]^2K_A^2\gamma+8[L]^3K_A^3\gamma^3\mu+[L]^4K_A^4\gamma^6\mu^4\nu}\qquad[3]$$

$$p'_3=\frac{8[L]^3K_A^3\gamma^3\mu}{2+32[L]K_A+24[L]^2K_A^2\gamma+8[L]^3K_A^3\gamma^3\mu+[L]^4K_A^4\gamma^6\mu^4\nu}\qquad[4]$$

$$p'_4=\frac{[L]^4K_A^4\gamma^6\mu^4\nu}{2+32[L]K_A+24[L]^2K_A^2\gamma+8[L]^3K_A^3\gamma^3\mu+[L]^4K_A^4\gamma^6\mu^4\nu}\qquad[5]$$

And the normalized current potentiated by the activator can be written as

$$\Delta I/\Delta I_{\max}=ap'_1+bp'_2+cp'_3+dp'_4\qquad[6]$$

Eq. 6 is a concentration-response equation for the mixture channel, which can be used to fit the experiment data measured by recording the steady state whole cell current as a function of activation concentration in solution. Note that the terms in the numerators and denominators of Eqs. 1-5 are quite similar to those of Eqs. 18-22 (main text), respectively. The difference between these two set of equations is the coefficient before each term, which reflects the microstate multiplicity of each configuration (CF1-CF4) for the two channels (WT and heterozygous).

Mathematical foundation of the model fitting well the experimental data

Eq. 7 is the Hill equation that is widely used to fit the concentration-response data for a chemical to activate an ion channel.

$$\frac{\Delta I}{\Delta I_{\max}}=\frac{1}{\left\{1+\left(\frac{EC_{50}}{[L]}\right)^H\right\}}\qquad[7]$$

where EC_{50} is the concentration of an activator at which $\Delta I/\Delta I_{max}$ is 50% of its maximum value), H is the Hill coefficient, and $[L]$ is the concentration of the activator.

From a simple mathematic derivation, we may obtain the second derivative of $\Delta I/\Delta I_{max}$ with respect to ln[L]

$$\frac{d^2}{d\left(\ln[L]\right)^2}\left(\frac{I}{I_{\max}}\right)=\left(EC_{50}\right)^H[L]\frac{H^2\left[L\right]^{H-1}\left\{\left(EC_{50}\right)^H-\left[L\right]^H\right\}}{\left\{\left[L\right]^H+\left(EC_{50}\right)^H\right\}^3} \qquad [8]$$

Eq. 8=0 has only one real root that is large than 0, i.e. [L]=EC_{50}. Accordingly, the concentration-response curve fitted by Hill equation is sigmoid and has only one inflection point at [L]=EC_{50}.

Both Eq. 23 (main text) and Eq. 6 indicate that our model can be express as

$$\frac{\Delta I}{\Delta I_{\max}}=\frac{\sum_{i=1}^{4}a_i\left[L\right]^i}{\sum_{j=0}^{4}b_j\left[L\right]^j} \qquad [9]$$

where a_i and b_j are constants expressed by the eight parameters, {K_A, γ, μ, v; a, b, c, d}, and i and j. We may also obtain the second derivative of $\Delta I/\Delta I_{\max}$ with respect to ln[L] for Eq. 9,

$$\frac{d^2}{d\left(\ln[L]\right)^2}\left(\frac{\Delta I}{\Delta I_{\max}}\right)=\frac{\sum_{m=1}^{15}a'_m\left[L\right]^m}{\sum_{k=0}^{16}b'_n\left[L\right]^n} \qquad [10]$$

where a'_m and b'_n are constants expressed by a_i, b_j, m and n. Obviously, Eq. 10=0 might have multiple real roots that are larger than 0, indicating that $\Delta I/\Delta I_{\max}$-Log[L] plot described by our models could have multiple inflection points, and thereby could fit well the experimental data. For example, Hill equation cannot fit the concentration response data for ztz240 potentiating the heterozygous KCNQ2 channel composed of WT subunits and F137A mutants because the trace of the experimental data is a bi-sigmoid curve and has two inflection points. Whereas our model fits well the experimental data with SSE= 0.0056 and R^2=0.99326 (see Figure 3(b)).

Definition of equations of statistical measures for the evaluation of fittings:

$$SSE=\sum_{i=1}^{n}\omega_i\left(y_i-\hat{y}\right)^2 \qquad [11]$$

$$R-square=\sum_{i=1}^{n}\omega_i\left(\hat{y}_i-\bar{y}\right)^2/\sum_{i=1}^{n}\omega_i\left(y_i-\bar{y}\right)^2 \qquad [12]$$

Here y_i is the observed data value, $\hat{y}_i$ is the predicted value from the fit and $\bar{y}$ is the average observed data. ω_i is the weighting applied to each data point, and $\omega_i = 1$ in this work.

REFERENCES

Li P, Chen Z, Xu H, Sun H, Li H, Liu H, *et al.* (2013). The gating charge pathway of an epilepsy-associated potassium channel accommodates chemical ligands. Cell Res **23:** 1106-1118.

Supplementary Figures and Legends

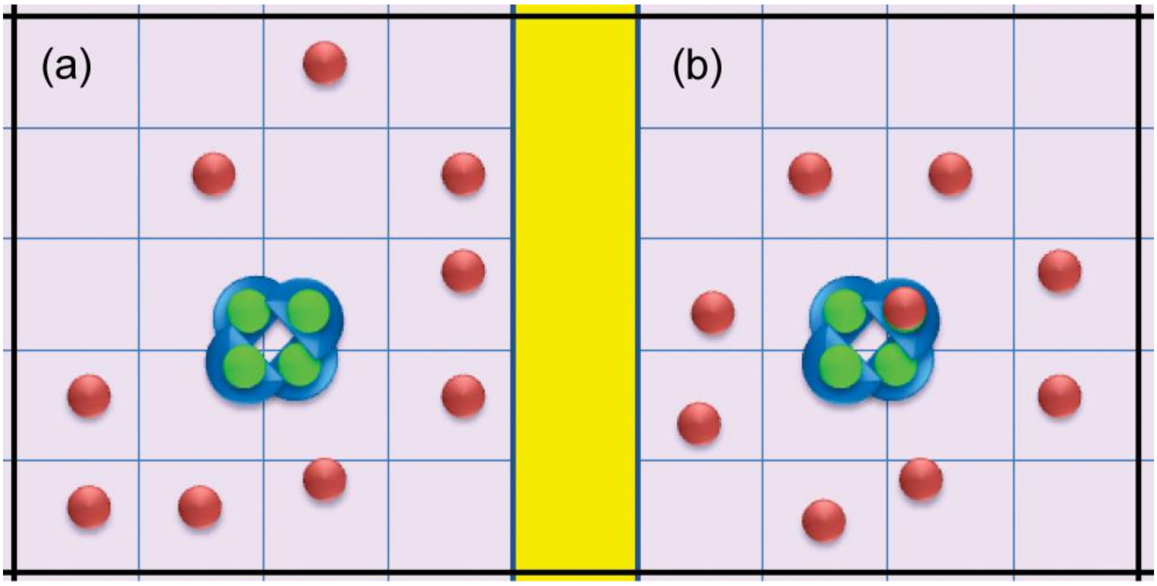

Figure S1. Lattice model for ztz240-KCNQ interaction. (a) An example for the microstates before ligand binding to channel; and (b) An example for the microstates after one ligand binding to the channel.

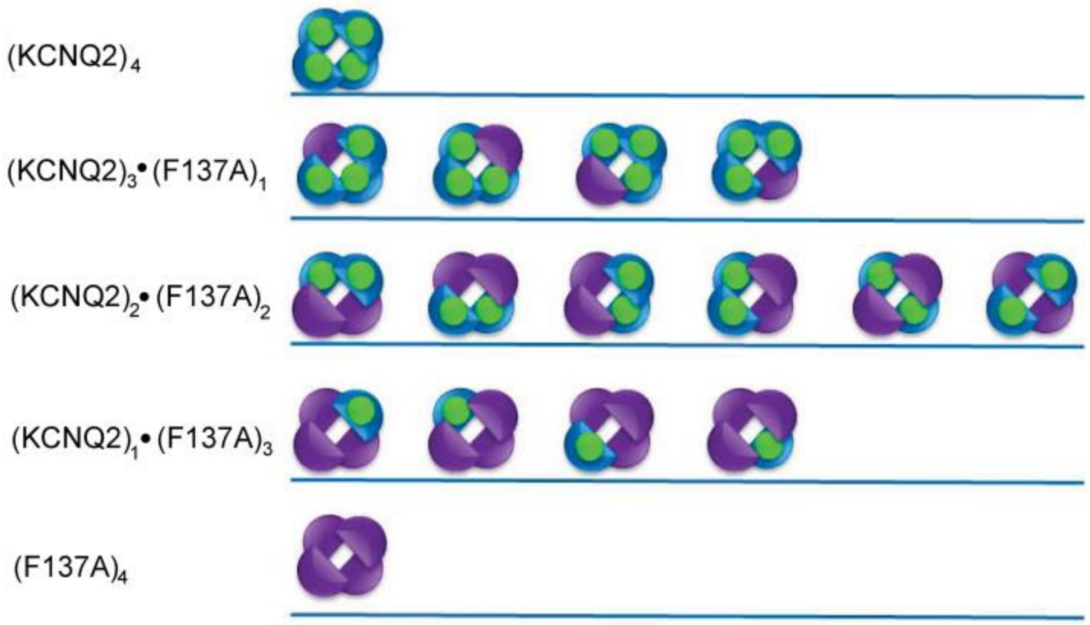

Figure S2. Possible combinations and corresponding microstates for the wild-type subunit and F137A mutant subunit to form tetramer channel. Wild-type subunits and F137A mutant subunits are represented by blue and purple semicircles, respectively. The green balls represent binding sites for activators.

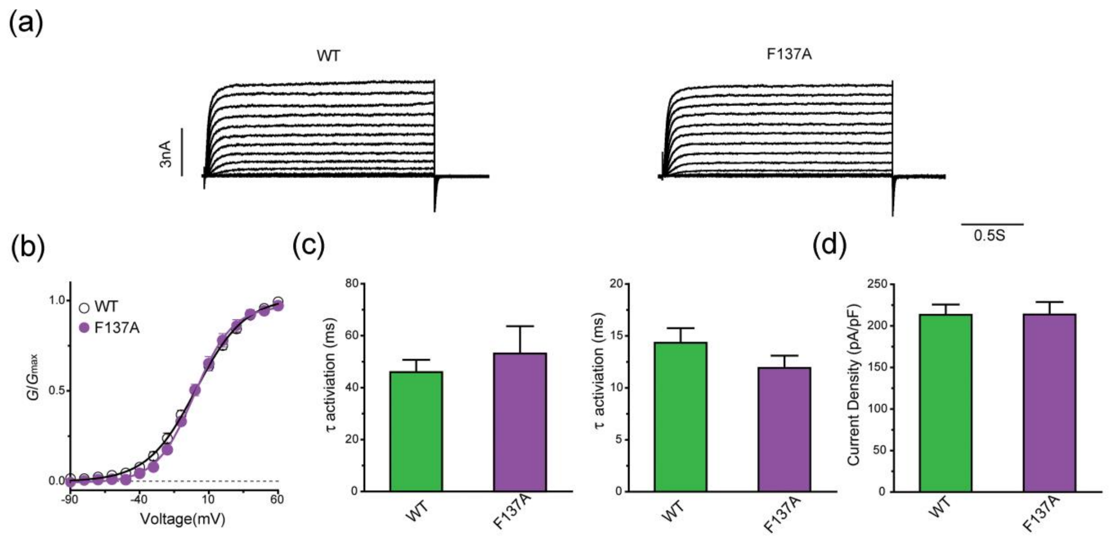

Figure S3. Electrophysiological assay results of the heterozygous channel. (a) The representative current traces of KCNQ2 WT and F137A mutant. The holding potential is -80 mV and the currents are elicited by depolarization steps from -90 mV to 60 mV with 10 mV increment; (b) The *G-V* curve of KCNQ2 WT and F137A channels. The $V_{1/2}$ value of each channel is 0.72 ± 0.98 (n = 9) and -0.37 ± 0.92 mV (n = 8), respectively; (c) The left panel is the activation time constants of each channel. The right panel is the deactivation time constants of each channel; and (d) The current density of each channel is 213.2 ± 12.4 (n = 5) and 213.7 ± 15.0 pA/pF (n = 5), respectively.

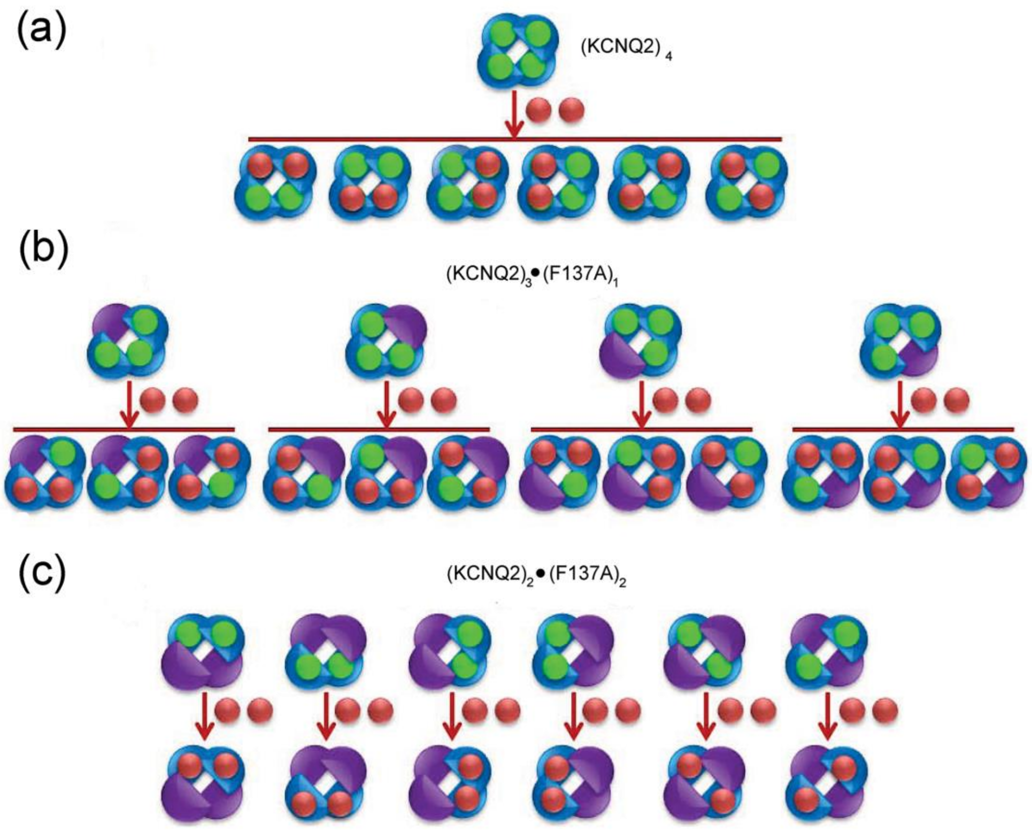

Figure S4. The twenty four microstates for two activators binding to the mixture channels (CF2). (a) The six microstates of the activator bind to the two binding sites of WT channel; (b) Two activators binding with the four microstates of $(KCNQ2)_3 \bullet (F137A)_1$ channel produce twelve binding microstates; and (c) The six binding microstates for two activators bind to the six microstates of $(KCNQ2)_2 \bullet (F137A)_2$ channel. Wild-type subunits and F137A mutant subunits are represented by blue and purple semicircles, respectively. The green balls represent binding sites for activators, and the wine balls indicate the activators.
